# Periodontal Tissue Regeneration Using Syngeneic Adipose‐Derived Stromal Cells in a Mouse Model

**DOI:** 10.5966/sctm.2016-0028

**Published:** 2016-09-16

**Authors:** Mathieu Lemaitre, Paul Monsarrat, Vincent Blasco‐Baque, Pascale Loubières, Rémy Burcelin, Louis Casteilla, Valérie Planat‐Bénard, Philippe Kémoun

**Affiliations:** ^1^ Department of Biological Sciences, Dental Faculty, Toulouse University Hospital, University of Toulouse, Toulouse, France; ^2^ CNRS ERL 5311, EFS, INPENVT, INSERM U1031, UPS, STROMALab, University of Toulouse, Toulouse, France; ^3^ Department of Anatomical Sciences and Radiology, Dental Faculty, Toulouse University Hospital, University of Toulouse, Toulouse, France; ^4^ UMR1048, I2MC, UPS, INSERM, University of Toulouse, Toulouse, France

**Keywords:** Mesenchymal stromal cells, Mesenchymal stem cell transplantation, Mice, Periodontitis, Regenerative medicine, Subcutaneous fat

## Abstract

Current treatment of periodontitis is still associated with a high degree of variability in clinical outcomes. Recent advances in regenerative medicine by mesenchymal cells, including adipose stromal cells (ASC) have paved the way to improved periodontal regeneration (PD) but little is known about the biological processes involved. Here, we aimed to use syngeneic ASCs for periodontal regeneration in a new, relevant, bacteria‐induced periodontitis model in mice. Periodontal defects were induced in female C57BL6/J mice by oral gavage with periodontal pathogens. We grafted 2 × 10^5^ syngeneic mouse ASCs expressing green fluorescent protein (GFP) (GFP+/ASC) within a collagen vehicle in the lingual part of the first lower molar periodontium (experimental) while carrier alone was implanted in the contralateral side (control). Animals were sacrificed 0, 1, 6, and 12 weeks after treatment by GFP+/ASC or vehicle graft, and microscopic examination, immunofluorescence, and innovative bio‐informatics histomorphometry methods were used to reveal deep periodontium changes. From 1 to 6 weeks after surgery, GFP+ cells were identified in the periodontal ligament (PDL), in experimental sites only. After 12 weeks, cementum regeneration, the organization of PDL fibers, the number of PD vessels, and bone morphogenetic protein‐2 and osteopontin expression were greater in experimental sites than in controls. Specific stromal cell subsets were recruited in the newly formed tissue in ASC‐implanted periodontium only. These data suggest that ASC grafting in diseased deep periodontium, relevant to human pathology, induces a significant improvement of the PDL microenvironment, leading to a recovery of tooth‐supporting tissue homeostasis. Stem Cells Translational Medicine *2017;6:656–665*

**Significance Statement**

Human periodontitis is a chronic, highly prevalent infectious disease characterized by the loss of both soft and hard tissues supporting the teeth. Current available treatments are insufficient, associated with a high degree of variability in clinical outcomes. The data in this study suggest that adipose‐derived mesenchymal stromal/stem cell (ASC) grafting in diseased deep periodontium, relevant to human pathology, promoted regeneration of deep periodontium, both in quantity and in quality, in comparison with controls. Even if mechanisms underlying periodontal regeneration by exogenous mesenchymal stromal cells are yet to be understood, this study brought to light new data regarding periodontal pocket regeneration induced by ASCs in mice.

## Introduction

Periodontitis is a chronic immuno‐infectious disease, characterized by loss of the tissues supporting the teeth, and leading to or aggravating systemic disorders such as diabetes, polyarthritis, or atherosclerosis 1. The defects result from a local homeostasis disruption caused by both the virulence of a periodontal pathogenic microflora 2 and an inappropriate immune response 3, 4. From a pathophysiology point of view 3, 4, the destruction of deep periodontium tissues (i.e., root cementum, periodontal ligament [PDL], and alveolar bone) induces the formation of crevices called “periodontal pockets” between the tooth root and its bony socket 5, leading to tooth loss.

Periodontal regeneration aims to restore both the architecture and function of tooth supporting tissues through the recruitment and activation of endogenous progenitors, especially those expressing CD146 markers 5, leading to renewal of the connective attachment underlying the new junctional epithelium. The restitution of dense connective fibers of PDL, anchored between the newly formed alveolar bone and root cementum, is critical for the long‐term prognosis 6, 7. A broad range of periodontal regenerative procedures has been proposed, including guided tissue regeneration, enamel matrix‐derived proteins, platelet‐rich plasma, and bone graft, but these procedures have been reported to lack efficiency and mainly result in incomplete defect reconstruction and poor reproducibility 8, 9. Persistence of low‐grade inflammation and infection, poor dental plaque control, blood clot stability, and systemic diseases may be involved in these unpredictable outcomes by preventing the activities of periodontal progenitors in situ 10.

Regenerative treatment of connective tissues therefore aims to create a microenvironment suitable for the migration, proliferation, and commitment of endogenous mesenchymal progenitors toward specific differentiation cell phenotypes involved in the synthesis of extracellular matrix components, such as bone morphogenetic proteins (BMP) or osteopontin (OPN) 6. Recent advances in regenerative medicine and the biology of mesenchymal stem cells have paved the way for new strategies based on tissue engineering 11. By their capacity to differentiate and acquire different phenotypes, to be stimulated by the local microenvironment, and to exhibit paracrine potential (e.g., mitogenic, angiogenic, antiapoptotic, immunomodulatory factor), exogenous mesenchymal stromal cells (MSCs) would favor the production of new tissues, by their own action or by stimulating the activity of endogenous progenitors 12, 13. MSCs can potentially be isolated from almost all organs 14 and are commonly purified from bone marrow, adipose tissue, and umbilical cord. For many reasons related to safety in tissue sample processing, access to cell sources, and availability, adipose‐derived mesenchymal stromal cells (ASCs) are expected to be a valuable source of cells and are being increasingly tested at the clinical level 15, 16.

A previous systematic review of the literature demonstrated the efficacy and safety of oral or extraoral MSCs (including ASCs) to regenerate periodontal tissues 17, but most of the studies were performed on poorly relevant defect models 17. Animal periodontal defects were usually induced mechanically using dental burs, with or without additional procedures (ligature or impression paste to favor bacterial colonization) 17, 18, 19 that did not create lesions or a tissue environment close to the pathophysiology of periodontitis.

In this study, we aimed to avoid such limitations by using a model with periodontal lesions induced by oral gavage with periopathogens, which led to periodontal defects relevant to human pathophysiology 17, 18. In this context, we investigated the use of syngeneic ASCs that expressed the green fluorescent protein (GFP) for in situ tracking and pointed out their ability to enhance deep periodontal tissue wound healing using classic and innovative bioinformatics measurements.

## Materials and Methods

### Periodontitis Mouse Model

The periodontitis model in mice was induced, as has already been described 20. This protocol was in accordance with the ARRIVE guidelines for reporting animal research 21. All procedures performed on mice were approved by the local ethics committees of Toulouse University Hospital and INSERM under the authorization number C3155507. C57BL6/J wild‐type female mice (Charles River, L'Arbresle, France, http://www.criver.com) were group‐housed (five per cage) in a specific pathogen‐free controlled environment with inverted 12‐hour daylight cycle in our animal facilities. Drinking water was supplemented with sulfamethoxazole (200 mg/5 ml) and trimethoprime (40 mg/5 ml) 10 days before bacterial oral gavage at a daily dose of 95 mg/kg.

Under isoflurane anesthesia at 8 weeks of age, the mice received 1 ml of a mix of 10^9^ colony‐forming unit of *Porphyromonas gingivalis* (ATCC 33277), *Fusobacterium nucleatum*, and *Prevotella intermedia*, as has been previously identified 18, in 2% carboxymethylcellulose in the molar regions. This step was repeated 4 times a week for 1 month to induce periodontal lesions.

### Isolation of GFP+ ASCs

Transgenic C57BL6/J mice constitutively expressing GFP were anesthetized by intraperitoneal administration of 100 mg/kg ketamine (Merial, Gerland, France, http://merial.com) and 10 mg/kg xylazin (Bayer, Puteaux, France, https://www.bayer.fr). Inguinal subcutaneous adipose tissues were processed as previously described to isolate ASCs 22. Briefly, inguinal adipose tissues were digested at 37°C for 45 minutes in phosphate‐buffered saline (PBS) containing 2% bovine serum albumin and 2 mg/ml collagenase 1 (Sigma‐Aldrich, St. Louis, MO, https://www.sigmaaldrich.com), filtrated at 25 µm, then centrifuged at 600 *g* for 10 minutes, to remove mature adipocytes. Red blood cells were lysed into buffer containing 140 mM NH_4_Cl and 20 mM Tris for 5 minutes at 4°C. Cells were centrifuged at 600 *g* for 5 minutes, and the vascular stromal fraction was seeded at 30 × 10^3^ cells per square centimeter in Dulbecco's modified Eagle's medium‐F12, supplemented with 10% newborn calf serum, 0.25 µg/ml amphotericin, 100 µg/ml streptomycin, and 100 UI/ml penicillin, and maintained in a 5% CO_2_ atmosphere.

### Characterization of ASC From GFP+ Mice

The ASC phenotype (passage 1) was confirmed by flow cytometry 23 using fluorescent‐ labeled anti‐SCA‐1 antibodies as a positive marker and anti‐CD45 and anti‐CD31 antibodies as negative markers (supplemental online Table 1) (BD Biosciences, East Rutherford, NJ, https://www.bdbiosciences.com). Exclusion of 4′,6‐diamidino‐2‐phenylindole was used for cell viability assessment. Fluorescence‐activated cell sorting (FACS Fortessa and FACS Diva software; BD Biosciences) revealed that 97% of ASC were positive for GFP (supplemental online Fig. 1A, 1B). Additionally, ASC/GFP+ were submitted to adipogenic or osteogenic media for 7 and 14 days. Multilineage differentiation was confirmed using quantitative polymerase chain reaction by Osterix, alkaline phosphatase 2, runt‐related transcription factor 1, adipocyte fatty acid‐binding protein, lipoprotein lipase, adiponectin, and peroxisome proliferator‐activated receptor‐γ expression, respectively (details on primers are found in supplemental online Table 1). Results are provided in supplemental online Figure 1C.

### Cell Grafting Into Mouse Periodontium

At 80% confluence, GFP+/ASC (passage 1) were trypsinized, counted, washed once in PBS, then used for transplantation. A gingival lingual flap was performed under binocular microscopy in the first lower molar region. A split mouth design was used: on one side, 2 × 10^5^ ASCs were applied in 20 µL of physiologic serum using 3 mm^3^ 2% type I collagen as the carrier, and the other side was used as a control and treated with the vehicle only (adapted from preliminary results and Tobita et al. 24). A total of 24 mice (48 periodontal defects) were used, distributed over four time points (0, 1, 6, and 12 weeks).

### Optical Microscopic Examination and Measurements

At the end of each time interval, mice were anesthetized and sacrificed by cervical dislocation; mandibles were collected, fixed in 4% formaldehyde, embedded in paraffin, then cut at 4 µm using a microtome (Jung 2055 Autocut, Leica Biosystems, Wetzler, Germany, http://www2.leicabiosystems.com). Sections were stained with Masson's trichrome and photographed under a light microscope equipped with a Nikon CoolPix 4500. Bone regeneration was assessed by measuring the distance between the cemento‐enamel junction (CEJ) and the top of the alveolar crest. A frame of 1,000 pixels^2^ (px^2^) surface area, representative of the cementum defect after 4 weeks of periopathogen infection, was drawn downward from the CEJ to the remaining cementum. An example of cementum measurement in an animal at 0 weeks (day of surgery) is provided in supplemental online Figure 2. The number of vessels inside the PDL was also counted. Each measurement was performed twice (blinded to previous assessment), on at least five sections per periodontal defect, and the mean of these five measurements was considered.

### Immunofluorescence Analyses

Immunofluorescence analyses were used to investigate the distribution of mineralized tissue markers (BMP‐2 and OPN), vessels (CD31), and connective progenitor subsets (supplemental online Table 1). Because endogenous GFP expression was too weak (data not shown) to highlight implanted cells, a rabbit alexa‐488 anti‐GFP was used to localize grafted GFP+/ASC (supplemental online Table 1). Paraffin was removed with xylene, and sections were rehydrated using a descending ethanol series. For the detection of intracellular markers only, permeabilization was performed first, using Triton ×100 in PBS at 0.1% for 15 minutes. Antigens were unmasked by incubation in citrate buffer (10 mM, pH 6.0) in an 80°C water bath for 20 minutes. Saturation of nonspecific sites was achieved by incubating the sections for 15 minutes at room temperature (RT) in PBS containing 5% normal serum from the same species as the host of the secondary antibody. Primary antibodies were used at the specified concentration (supplemental online Table 1) for 2 hours in a humidified chamber at RT for surface markers, or overnight at 4°C for intracellular targets. Slides were rinsed three times for 5 minutes in PBS containing 0.2% Tween 20. Secondary antibodies were then used at the specified concentration (supplemental online Table 1) for 1 hour at RT, then washed. Slides were mounted using ProLong© containing Hoechst (Thermo Fisher Scientific, Waltham, MA, https://www.thermofisher.com) and photographed using a confocal microscope (Zeiss LSM 780; Carl Zeiss AG, Oberkochen, Germany, http://www.zeiss.com).

### Hough Transform Analyses to Quantify Entropy of PDL Fibers

A measure of the PDL fiber architecture was achieved using the Hough transform (HT). Images were first oriented vertically using a line tangent to the root. The red component of the color image was kept to better visualize the fibers. We submitted the oblique and horizontal fibers of PDL to HT using Matlab 2012 software (Mathworks, Meudon, France, http://www.mathworks.com). Supplemental online Figure 3 summarizes the processing steps. Using HT, we drew lines corresponding to the main directions of detected fibers. The probability for each angular direction was derived from the Hough transform matrix and plotted as a histogram distribution. The entropy of this distribution was computed, which provided a statistical measure of randomness. A decrease of this parameter means that fibers are better orientated 25.

### Statistical Analysis

An analysis of variance with some random effect was used to determine whether a difference between the experimental side and the control side could be detected in noncolonized tissues, at 0 weeks (baseline), 6 weeks, or 12 weeks. Parameters in the experimental side were compared with the corresponding side of noncolonized and 0‐week mice, corrected by multiple comparisons using the Bonferroni adjustment. The level of significance was set to 0.05. Graphics and statistics were performed using Stata 13.1 (StataCorp, College Station, TX, http://www.stata.com).

## Results

### Grafted GFP‐Expressing ASC Were Identified in PDL From 1 to 6 Weeks After Surgery

**Figure 1:**
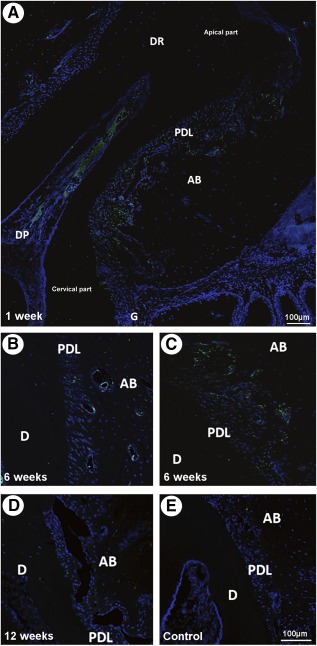
Localization of the grafted green fluorescent protein (GFP)+/adipose stromal cells (ASCs). Cells were tracked by immunofluorescence. **(A):** GFP+/ASC were identified in the experimental, periodontium‐implanted site at 1 week, not only close to the wound bed near the cervical part but also toward the apical part of the periodontal ligament (PDL) and surrounding the ligament and alveolar bone blood vessels. **(B, C):** GFP+/ASC localization surrounding PDL and alveolar bone blood vessels **(B)** and in the apical part of the PDL **(C)**. **(D):** Undistinguishable GFP+/ASC in the grafted side at week 12. **(E):** Undistinguishable cells in vehicle‐only treated control sites. Scale bar = 100 µm. Abbreviations: AB, alveolar bone; D, dentin; DP, dental pulp; DR, dental root; G, gingiva; PDL, periodontal ligament.

During the course of the periodontal wound healing, we used immunofluorescence (IF) microscopy to follow the distribution and fate of grafted GFP+/ASC (Fig. 1A–1E). From 1 to 6 weeks after surgery, ASC were localized only in the experimental site close to the wound bed near the CEJ, toward the apical part of the PDL, and surrounding PDL and alveolar bone blood vessels (Fig. 1 A–1C). Cells expressing the GFP marker were almost undetectable at ASC‐implanted sites after 12 weeks (Fig. 1D) and at control‐treated sites (Fig. 1E).

### ASC Grafting Enhanced Cementum Regeneration, PDL Fiber Organization, and Number of Vessels

**Figure 2:**
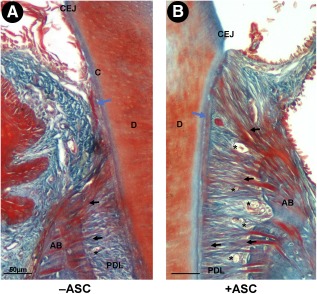
Adipose stromal cell (ASC) graft improved deep periodontium regeneration. Histological section of mouse deep periodontium 12 weeks after vehicle **(A)** or ASC **(B)** grafting. Cementum deposition (blue arrow), PDL fiber organization (black arrows), and number of vessels (black stars) had increased in the experimental condition. Scale bar: 50 µm. Abbreviations: AB, alveolar bone; CEJ, cemento‐enamel junction; D, dentin; PDL, periodontal ligament.

**Figure 3:**
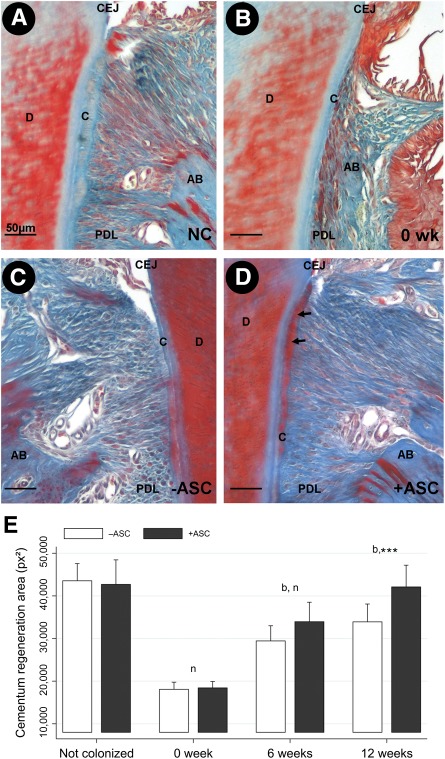
Adipose stromal cell (ASC) graft improved cementum regeneration. Histological sections of healthy **(A)** and diseased cementum before treatment (0 week) **(B)** and with 12‐week vehicle‐only treated tissue **(C)** or ASC‐treated tissue **(D)**. **(E):** Histomorphometry analysis of cementum deposition. The area of cementum was measured in square pixels on the control side (white bars) and the ASC‐grafted side (black bars). Twelve weeks after grafting, the cementum was rescued in the ASC‐treated side only. Scale bars: 50 µm. ∗∗∗, *p* < .001, indicating a significant difference between treatment and control sides. Abbreviations: AB, alveolar bone; b, indicates a significant difference in the treatment side between each time point and 0 week (baseline); C, cementum; CEJ, cemento‐enamel junction; D, dentin; n, indicates a significant difference of the treatment side at each time point and not colonized; NC, not colonized; PDL, periodontal ligament; px, pixel; wk, week.

**Figure 4:**
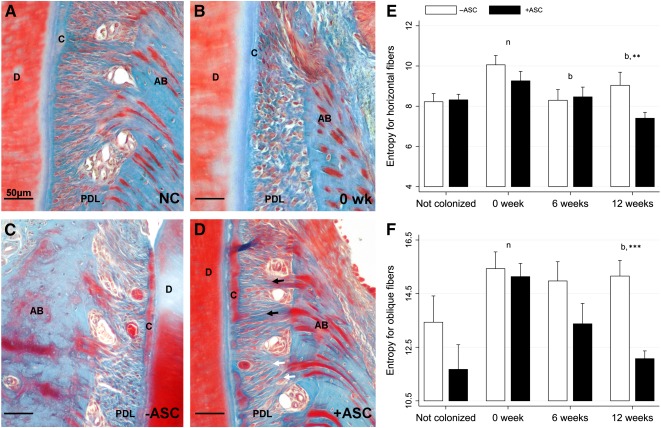
Adipose stromal cell (ASC) graft improved periodontal ligament (PDL) fiber reorganization. **(A–D):** Histological sections of healthy **(A)** and diseased PDL fibers before treatment (0 week) **(B)** and 12‐week vehicle treated **(C)** or ASC treated **(D)**. Black arrows indicate horizontal fibers and white arrows oblique fibers.** (E–F):** Histomorphometry analysis of PDL. Twelve weeks after treatment, the entropy calculated from the Hough transform was significantly lower in ASC‐grafted sites than in controls, for both horizontal **(E)** and oblique **(F)** fibers, and close to that of healthy PDL. Scale bar: 50 µm. ∗∗, *p* < .01; ∗∗∗, *p* < .001, indicating a significant difference between treatment and control sides. Abbreviations: AB, alveolar bone; ASC, adipose stromal cell; b, indicates a significant difference in the treatment side between each time point and 0 week (baseline); C, cementum; D, dentin; n, indicates a significant difference in the treatment side at each time point and not colonized; NC, not colonized; PDL, periodontal ligament; wk, week.

Overall, 12 weeks after treatment of diseased deep periodontium by ASC or vehicle, ASC‐grafted sites exhibited higher cementum deposition, enhanced periodontal fiber organization, with denser Sharpey's fibers, and an increase in PDL vascularization in comparison with controls (Fig. 2). From 6 weeks after treatment, cementum and PDL regeneration occurred in both control and experimental sites (Figs. 3, 4). Microscopic examination of 12‐week ASC‐treated periodontium showed that newly deposited cementum‐like tissue was thicker than at contralateral vehicle‐only grafted sites and was similar to healthy cementum (Fig. 3A–3D), as confirmed by histomorphometry. This demonstrated that the cementum thickness was entirely recovered only on experimental sides (Fig. 3E; *p* < .001). The amount of cementum regeneration increased over time and was significantly higher that at the starting point (0 week).

PDL fiber organization (orientation, length, and density) was stronger in the experimental side than the control side. Sharpey anchorage appeared denser and more homogenous in ASC‐ grafted PDL tissues than in controls. As for cementum regeneration, PDL microscopic appearance was close to that of healthy structures in experimental sites but not in vehicle‐only treated sites (Fig. 4A–4D). The Hough transform (HT) was used to quantify oblique and horizontal fiber organization by determination of the structure entropy (supplemental online Fig. 3). Twelve weeks after treatment, the entropy of both oblique and horizontal PDL fibers was significantly lower in experimental sites than in control sites, suggesting that the ASC graft enhanced periodontal connective attachment regeneration (Fig. 4E, 4F). Interestingly, fiber entropy time decay was confirmed, and fiber organization completely rescued in ASC‐ implanted sites only.

**Figure 5:**
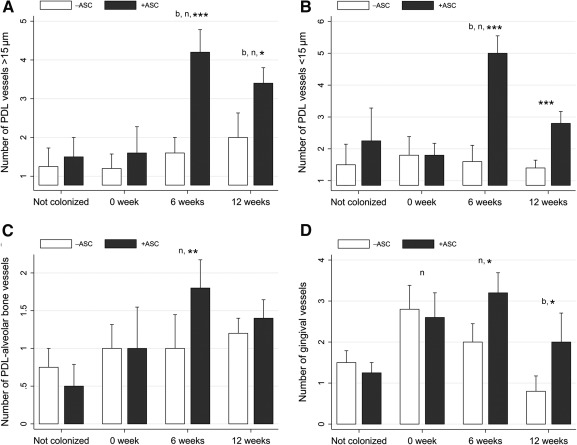
Adipose stromal cell (ASC) graft enhanced periodontal ligament (PDL) neovascularization. **(A–D):** Six weeks after treatment, the number of >50‐µm diameter PDL vessels **(A)**, <50‐µm diameter PDL vessels **(B)**, alveolar bone PDL vessels **(C)**, and gingival PDL vessels **(D)** was significantly higher in ASC‐treated sites than in vehicle‐treated sites. ∗, *p* < .05; ∗∗, *p* < .01; ∗∗∗, *p* < .001, indicating a significant difference between treatment and control sides. Abbreviations: ASC, adipose stromal cell; b, indicates a significant difference in the treatment side between each time point and 0 week (baseline); n, indicates a significant difference of the treatment side at each time point and not colonized; PDL, periodontal ligament.

The microscopic evaluation of PDL vascularization showed that grafting ASCs promoted a significant increase in the number of both small‐ and large‐diameter PDL vessels, from two‐ to fourfold, in comparison with control at 6 and 12 weeks (Fig. 5A, 5B), in PDL, in alveolar bone at 6 weeks (Fig. 5C), and in gingiva at 6 and 12 weeks (Fig. 5D). These observations were confirmed by CD31 distribution in controls and experimental periodontium at 6 weeks (supplemental online Fig. 4). Six weeks after treatment, the number of PDL vessels was greater than at the starting point and in healthy tissues but decreased later.

Taken together, these data indicate that cementum regeneration, PDL fiber organization and PDL vessel number were improved in experimental conditions in relation to control sites.

### Deep Periodontium BMP‐2 and OPN Expression Is Modified by ASC Graft

**Figure 6:**
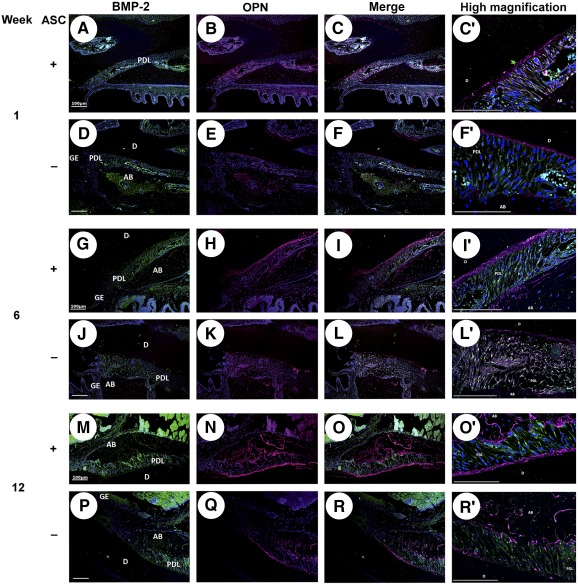
Effect of adipose stromal cell (ASC) graft on bone morphogenetic protein (BMP)‐2 and osteopontin (OPN) expression during periodontal healing. BMP‐2 (green; **A, D, G, J, M, P**) and OPN staining (magenta; **B, E, H, K, N, Q**), and their colocalization **(C, F, I, L, O, R)** 1, 6, and 12 weeks after deep periodontium grafting ±ASC. Six weeks after surgery, BMP‐2 expression extended toward the apical part of the periodontal ligament on the experimental side only. OPN expression underlined the cementum deposition and was clearly enhanced by ASC implantation in comparison with control. High magnifications of colocalization are displayed **(C′, F′, I′, L′, O′, R′)**. Cell nuclei are in blue. Scale bar: 100 µm. Abbreviations: AB, alveolar bone; ASC, adipose stromal cell; BMP‐2, bone morphogenetic protein‐2; D, dentin; GE, gingival epithelium; OPN, osteopontin; PDL, periodontal ligament.

To investigate the distribution of noncollagen matrix markers during the periodontal regeneration, we analyzed the change in BMP‐2 and OPN expression by immunofluorescence microscopy (Fig. 6). One week after treatment, BMP‐2 staining was mainly identified in the cervical part of the PDL in both vehicle‐ and ASC‐grafted tissues (Fig. 6A, 6D). Six weeks after surgery, BMP‐2 expression was stronger in ASC‐grafted sites than in control sites and extended toward the apical part of the PDL on the experimental side only (Fig. 6G, 6J). Twelve weeks after challenge, the expression of BMP‐2 had returned to normal in experimental and control sites (Fig. 6M, 6P).

During the course of periodontal tissue regeneration with or without ASC grafting, OPN expression underscored the cementum deposition and was clearly more enhanced in experimental sites than in controls. As for BMP‐2 expression, OPN staining highlighted the PDL reorganization (Fig. 6B, 6E, 6H, 6K, 6N, 6Q). PDL examination revealed OPN and BMP‐2 colocalization (Fig. 6C, 6C′, 6F, 6F′, 6I, 6I′, 6L, 6L′, 6O, 6O′, 6R, 6R′), but strong OPN deposition only sustained the PDL/cementum and PDL/alveolar bone interfaces in comparison with BMP‐2 staining.

### ASC Graft Impacted the PDL Expression of SCA‐1 and CD146 During Periodontal Healing

**Figure 7:**
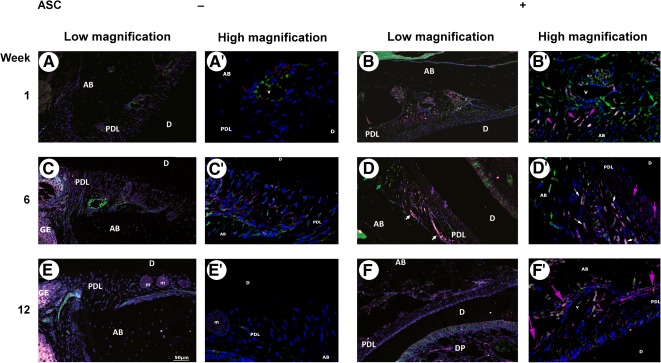
Adipose stromal cell (ASC) graft affected the periodontal ligament (PDL) expression of SCA‐1 and CD146 during periodontal healing. Colocalization of SCA‐1 (green) and CD146 (magenta) expression 1 week **(A, A′, B, B′)**, 6 weeks **(C, C′, D, D′)**, and 12 weeks **(E, E′, F, F′)** after deep periodontium grafting ±ASC. After 6 weeks, ASC periodontium implantation only clearly promoted the emergence of SCA‐1+/CD146− (green arrows) and SCA‐1+/CD146+ (white arrows) populations in perivascular locations, and an SCA‐1−/CD146+ (magenta arrows) subset indicating the cementum‐lining cells. Cell nuclei are in blue. Scale bar: 50 µm. Abbreviations: AB, alveolar bone; ASC, adipose stromal cell; D, dentin; DP, dental pulp; GE, gingival epithelium; m, epithelial cell rests of Malassez; PDL, periodontal ligament; v, vessel.

Next, we used IF to compare the expression of SCA‐1 and CD146, two surface markers for connective tissue progenitors, in healing periodontal tissues with or without ASC grafting (Fig. 7). SCA‐1 and CD146 expression in the PDL cell population were clearly modified by ASC implantation from 1 to 12 weeks after surgery, in comparison with vehicle‐only treated sites.

One week after treatment of mouse altered periodontium by syngeneic ASC, numerous distinct SCA‐1+/CD146− and SCA‐1−/CD146+ cell populations were localized in the alveolar bone side of the PDL, mainly surrounding blood vessels, whereas these cell subsets were hardly seen in control PDL (Fig. 7A, 7A′, 7B, 7B′). After 6 weeks, experimental sites exhibited SCA‐1−/CD146+ PDL cells underlining the putative cementoblast layer, while SCA‐1+/CD146− subsets remained located around PDL vessels. Interestingly, a transient perivascular SCA‐1+/CD146+ PDL cell population emerged at this stage and was completely lacking at control sites (Fig. 7C, 7C′, 7D, 7D′). Finally, in 12‐week challenged animals, SCA‐1 expression had almost entirely disappeared in PDL. The SCA‐1−/CD146+ PDL cell population remained highlighted in the PDL/alveolar bone interface in ASC‐grafted sites only (Fig. 7E, 7E′, 7F, 7F′).

## Discussion

Our data point out that syngeneic exogenous ASC may be very useful in periodontal regeneration 18. In a murine model relevant for human tooth‐supporting tissue pathophysiology and after ASC transplantation, we highlighted structural and functional changes occurring in deep periodontal tissues during the regeneration process. Moreover, our data show, for the first time, that ASC graft enhances all the deep periodontium healing: cementum regeneration as well as PDL organization, neocapillarization, and expression of progenitor/matrix markers.

A complete cementum recovery is crucial for sustaining a long‐term favorable outcome because it is essential for strong anchoring of Sharpey PDL fibers in the root and, thus, the maintaining of the tooth in its socket 26. Such cementum regeneration is clearly enhanced by ASC grafting, in line with a recent study in dogs 27. The improvement we report here of OPN and BMP‐2 expression on the experimental side confirms the positive effect of ASC grafting in cementum regeneration. Such an increase in OPN has been documented in a periodontal fenestration rat model in which PDL cells were grafted 28. This is consistent with the crucial role of OPN in the recruitment and maintenance of selective cells at the root surface 26. Moreover, OPN expression is critical for local innate immunity, inducing macrophage recruitment on site, and tissue remodeling 29. Indeed, the immunomodulatory properties of ASCs are frequently presented as an attractive and major effect to consider to modulating immune as well as inflammatory responses in vitro and in vivo, such effect being mediated by cytokine secretion and cell‐cell interaction. This close relationship suggests the existence of a dynamic and reciprocal regulation network between immune and mesenchymal stromal cells.

Fiber orientation and density encourage a nurturing role for cementum, a uniform distribution of masticatory forces, and a remodeling of the alveolar bone 30. The present results suggest that ASC use may enhance the formation of new functional PDL, by the increase of well‐oriented oblique and horizontal fibers 17. Moreover, BMP‐2 distribution was found to emphasize the reorganization and orientation of PDL fibers during the periodontal wound healing and was transiently upregulated in ASC‐treated sides in comparison with controls. In a rat fenestration model, regeneration of PDL fibers, well‐orientated perpendicularly to the root surface, was observed after bone marrow‐MSC grafting 31. Moreover, in an acute rat rotator cuff repair model, with special focus on the healing of the tendon‐to‐bone insertion, grafted ASC in a collagen carrier led to significantly more elastic and less scarred newly formed tissue than in control 32. The use of MSC for scarring in aged mice demonstrated increased wound tensile strength 33. Altogether, these data strongly suggest that ASC therapy enhances collagen fiber reorganization during PDL wound healing.

Because the blood supply is critical for optimal wound healing, we investigated the impact of ASC use in periodontal neoangiogenesis. As described in a murine skin wound healing model 34, an increase in the number of PDL vessels was shown at the cell‐treated site. Interestingly, both endogenous and grafted MSC were found to be located around blood vessels, as has been already reported 34, suggesting cross‐talk between mesenchymal progenitors and endothelial cells. ASC have been reported to stabilize endothelial cell networks by enhancing pericyte properties, thus improving vascular network formation 35, 36. Moreover, pericytes are suggested to share MSC features and may be involved in tissue regeneration/repair by differentiation toward specialized connective phenotypes 37, 38. However, the mechanisms by which ASC improve vascular network are still unknown.

Regeneration of connective tissue is mainly based on the activation of specific signaling pathways involved in the recruitment and mobilization of endogenous MSC in the wound bed 39. Whether grafted ASCs act through transdifferentiation or recruitment and commitment of local stem/progenitor cells remains under discussion. The current consensus based on experimental models and cell tracking argues that even if transdifferentiation events may occur, ASCs are more likely acting by supporting endogenous cell differentiation potentials 22, 40. Indeed, although CD146 and SCA‐1 proteins may be expressed by subsets of grafted murine ASC 37, 38, 41, the progressive fade‐out of grafted cells conversely to the increase of CD146 and SCA‐1 5, 42, 43 (osteocementogenic precursors marker) expression in PDL strongly suggests that implanted cells do not themselves differentiate toward specialized target phenotypes but rather induce a microenvironment suitable for progenitor recruitment from substratum. Thus, these data suggest that ASC graft in situ activities may be mediated via a paracrine effect, although their progressive phasing in implanted sites could also provide signaling for the local environment, as has been already reported 44, 45.

Six weeks after implantation, experimental sites exhibited SCA‐1 positive cells, some of which expressed CD146, surrounding blood vessels. Because SCA‐1 was shown to characterize undifferentiated mesenchymal pools 46, this paravascular cell population, mainly emerging in grafted sites, may be considered early periodontal precursors. Moreover, our results point out that a CD146+ SCA‐1− cell subpopulation is located under the cementoblast layer. This subset may be regarded as a predifferentiated population, already committed to the cementoblastic lineage 5. In line with this result, it was reported that the expression of the insulin‐like growth factor binding protein 6 can be enhanced in human periodontal ligament cell (hPDLC) by coculture with ASCs, which release appropriate trophic factors, thus supporting hPDLC differentiation into mineralized tissue‐forming cells, such as osteoblasts and cementoblasts 47. Overall, these data suggest that ASC grafting may enhance the recruitment and commitment of endogenous periodontal progenitors that correlate with the promotion of deep periodontal tissue regeneration, as was previously reported in other applications of ASC therapy 48, 49, 50, 51, 52, 53.

Surprisingly, we did not find any marked effect of ASC in alveolar bone regeneration (supplemental online Fig. 5). Alveolar bone regeneration by cell therapy is controversial and depends on the animal species, the defect designs, and the sources and carriers of grafted cells. Conversely to infrabony defects, well‐known to be regenerated with high predictability, our periodontitis‐pathogen‐induced alveolar bone defect model had a horizontal shape. Thus, we can hypothesize that the morphology of these defects could be unsuitable for assessing the effect of ASC grafting in alveolar bone reconstruction.

Altogether, our data show, in a very relevant model of rodent periodontitis, that ASC grafting significantly supports the periodontal regeneration linked not only to enhanced cementum regeneration, as earlier reported 24 but for the first time enhanced PDL fiber organization and number of vessels, as well as specific progenitors and periodontal cell lineage marker expression. These data suggest that ASC‐cell grafting could be a future clinical therapy for periodontal disease.

## Author Contributions

M.L. and P.M.: conception and design, collection and assembly of data, data analysis and interpretation, manuscript writing; V.B.‐B.: conception and design, collection and assembly of data, data analysis and interpretation, final approval of manuscript; P.L.: conception and design, provision of study material or patients, collection and assembly of data, final approval of manuscript; R.B.: manuscript writing, language check; final approval of manuscript, financial support; L.C.: conception and design, financial support, data analysis and interpretation, manuscript writing, language check, final approval of manuscript; V.P.‐B.: conception and design, collection and assembly of data, manuscript writing, final approval of manuscript; P.K.: conception and design, administrative support, collection and assembly of data, data analysis and interpretation, manuscript writing, final approval of manuscript.

## Disclosure of Potential Conflicts of Interest

The authors indicated no potential conflicts of interest.

## Supporting information

Supporting Information
