# Supplementary material for: Periodontal Tissue Regeneration Using Syngeneic Adipose‐Derived Stromal Cells in a Mouse Model
Source: Stem Cells Transl Med. 2016 Sep 16;6(2):656–65. doi: 10.5966/sctm.2016-0028 (PMC5442818; doi:10.5966/sctm.2016-0028)
Supplement: Supplementary file 1 — Supporting Information [file SCT3-6-656-s001.pdf]

Supplemental Information - Kémoun et al.

Supplemental Figures

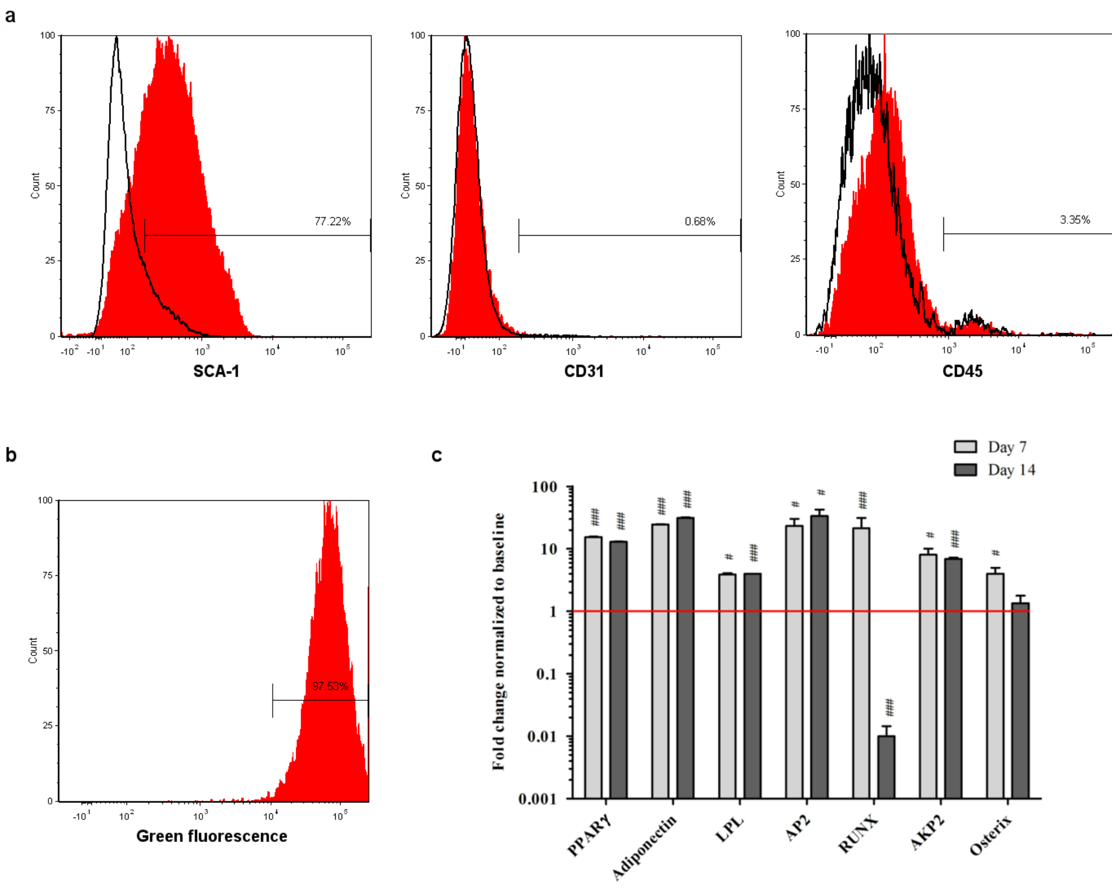

Fig. S1: Characterization of ASC from GFP+ mice. (a) Illustration of a flow cytometry analysis of CD31, CD45 and SCA-1 markers for DAPI negative (viable) ASC from GFP mice (red line). Black line: Isotype-matched negative control antibody. (b) Green fluorescence acquisition for ASC from GFP mice. (c) Fold change of Osterix, AKP2, Runx, AP2, LPL, Adiponectin, PPARγ gene expression compared to 36B4 and normalized to baseline values, 7 and 14 days-maintained in medium with osteogenic or adipogenic supplement. "#", "##" and "###" indicate a significant difference between treatment and control sides with $p<0.05$, $p<0.01$ and $p<0.001$ respectively.

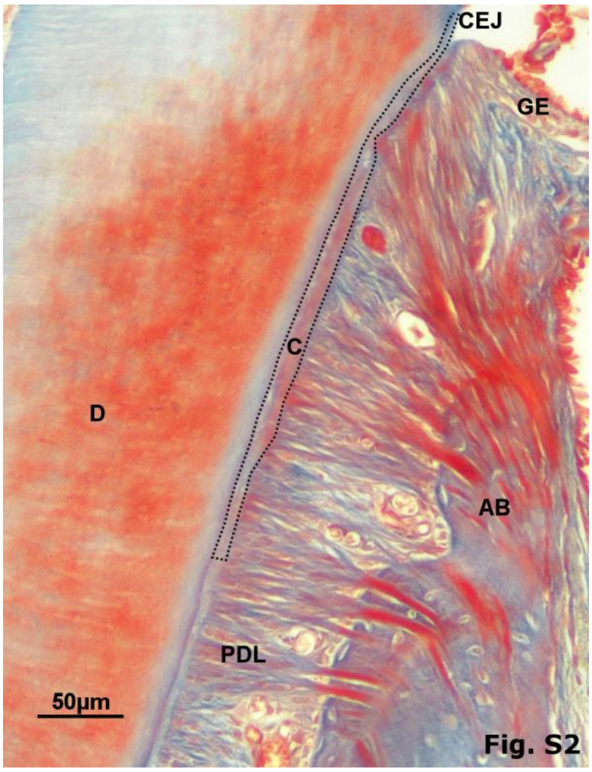

Fig. S2: Example of cementum measurement. In untreated periodontitis-induced mice (0 week, day 0 surgery), a frame of 1000 pixels' height was drawn downward the CEJ to the remaining cementum surface area, here in dotted lines, to quantify the cementum defect.

AB: alveolar bone, C: cementum, CEJ: cemento-enamel junction, D: dentin, GE: gingival epithelium, PDL: periodontal ligament.

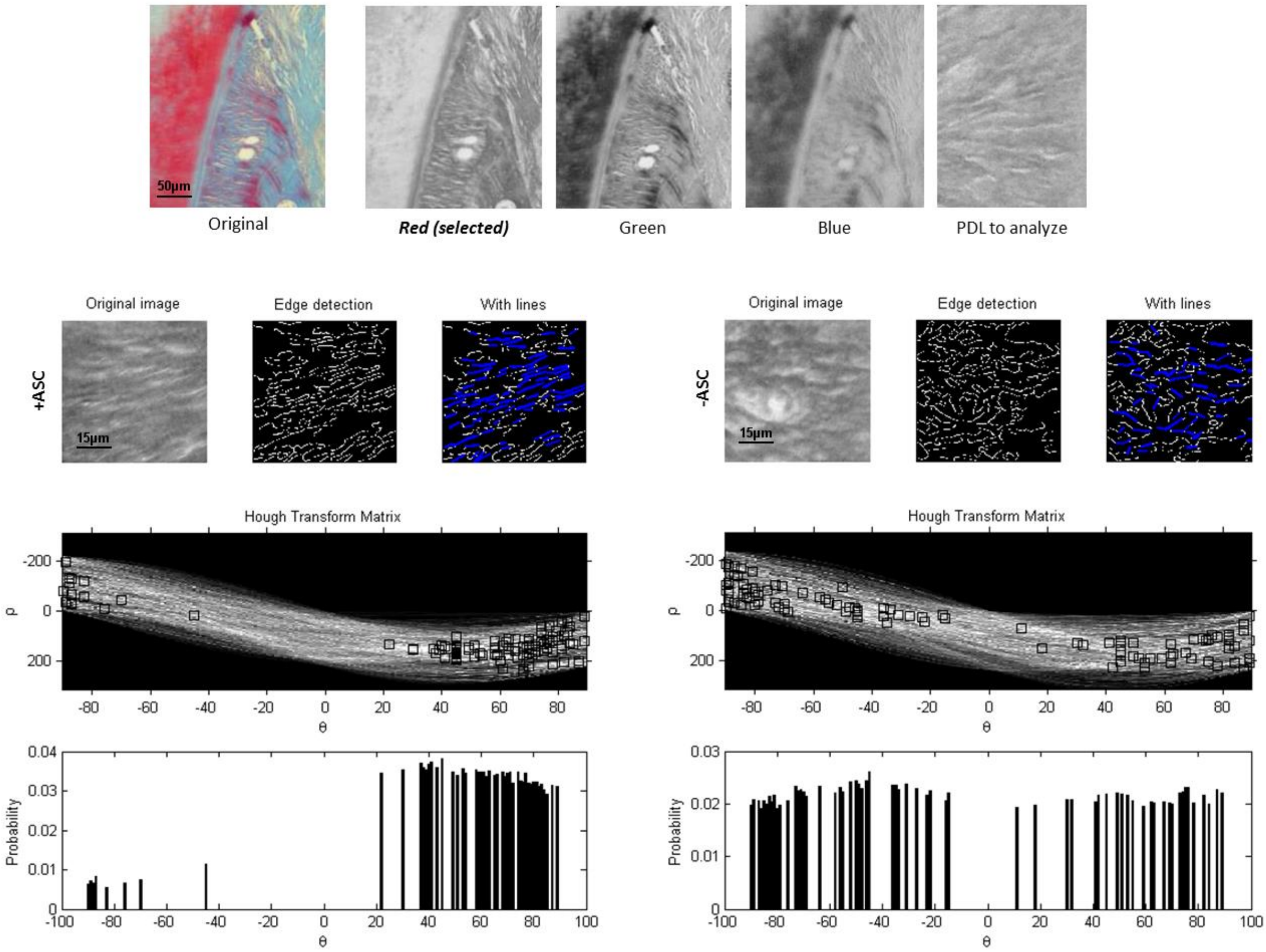

Fig. S3: Measure of the periodontal ligament organization by entropy. Red components of histological sections were extracted, edges were detected and the Hough Transform matrix was employed to select lines corresponding to the main directions of detected fibers. The probability for each angular direction was plotted as a histogram. The entropy of this distribution was computed, which provided a statistical measure of randomness.

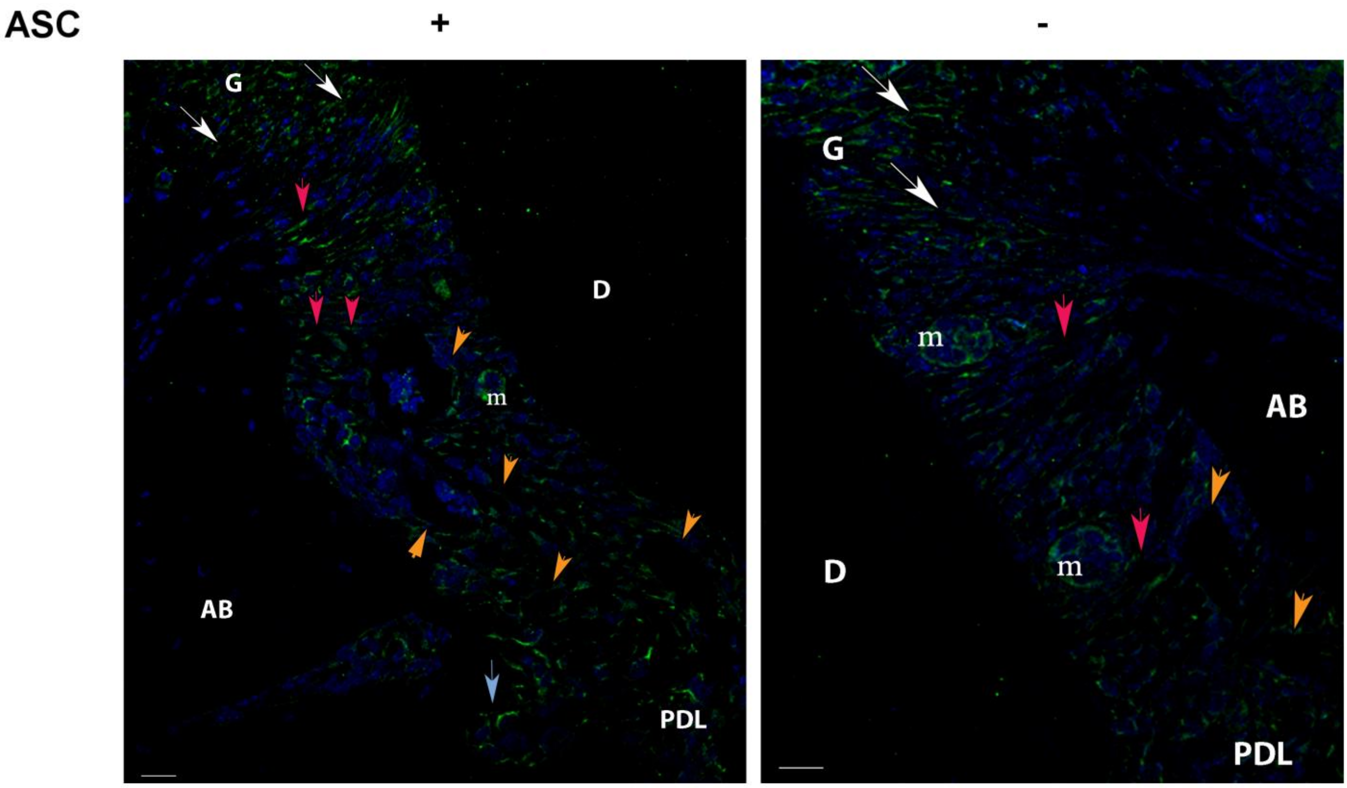

Fig. S4: CD31 expression in periodontal tissue, 6 weeks after grafting with (+) or without (-) ASC+/GFP. Gingiva (white arrows), small (red arrows), large (orange arrows) and AB (blue arrow) vessels are displayed; cell nuclei in blue. AB: alveolar bone, D: dentin, G: gingiva, m: epithelial cell rests of Malassez, PDL: periodontal ligament. Bar: 50 µm.

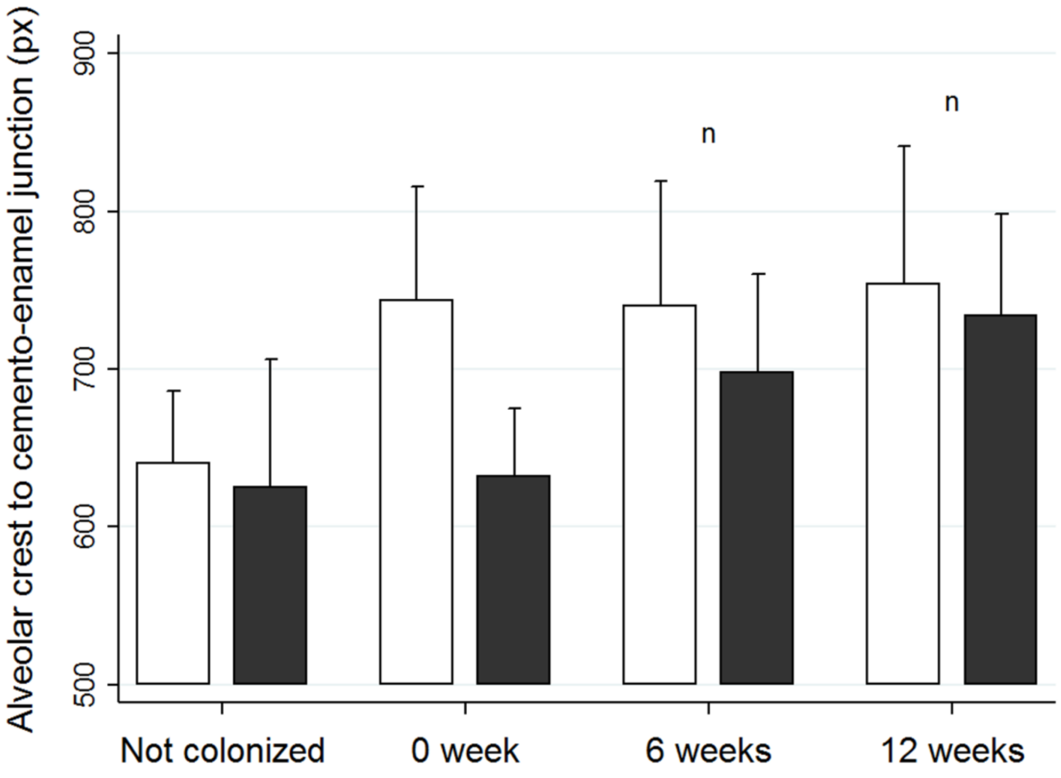

Fig. S5: No effect of ASC for alveolar bone regeneration. Results from histomorphometry analysis of alveolar bone. The distance between the alveolar crest and the cemento-enamel junction was measured in pixels in the control side (white bars) and the grafted side (gray bars). The “n” code indicated a significant difference of the treatment side between each time point and baseline and not colonized.

Supplemental Table

Antibodies for immunofluorescence		Supplier	Dosage (v:v)
Bone Morphoprotein (BMP-2)<br>Goat anti-mouse	Primary	Santa Cruz technologies	1:50
Osteopontin (OPN)<br>Rabbit anti-mouse	Primary	LF -175<br>Larry Fisher	1:400
SCA-1<br>Rat anti-mouse	Primary	BD Pharmingen	1:100
Rabbit anti-GFP Alexa 488	Primary	Invitrogen	1:75
CD146<br>Sheep anti-mouse	Primary	R&D systems	1:75
CD31 (PECAM-1)<br>Goat polyclonal M-20	Primary	Santa Cruz technologies	1:100
Swine anti-goat Alexa 488	Secondary	Invitrogen	1:200
Donkey anti-rabbit Alexa 594	Secondary	Invitrogen	1:150
Donkey anti-rat Alexa 594	Secondary	Invitrogen	1:200
Donkey anti-sheep Alexa 647	Secondary	Invitrogen	1:150
Goat anti-rat Alexa 488	Secondary	Invitrogen	1:200

Target for qPCR	Reverse	Forward
Osterix	GATCAGATCCCCATTGGACTTC	CCAGAGTTAAGGAGATTGGTGTTAGTAA
AKP2 - Alcaline phosphatase 2	GATTCGGGCAGCGGTTACT	CACCAATGTAGCCAAGAATGTCAT
Runx	TGGGTCCACACACCAACGCT	TCAAAATCACAGTCACCGC
AP2 - Adipocyte fatty acid-binding protein	GCCATGCCTGCCACTTTG	GATGCCTTGTGGGAACCTG
LPL<br>Lipoprotein lipase	CTCTTGGTTTGTCCAGTGT	ATCTGCAGAAGGGAAAGGACTC
Adiponectin	CCCTTCAGCTCCTGTCATTCC	TCCTGGAGAGGGAGAGAAAG
PPARγ	GCACCATGCTCTGGGTCAA	AGTGTGAATTACAGCAAATCTCTGTTTT
36B4 - Gene taken as reference	GGCTGACTTGGTTGCTTTGG	AGTCGGAGGAATCAGATGAGGAT

**Flow cytometry antibodies**	**Supplier**	**Clone**	**Conjugates**
CD31	BD Biosciences	MEC 13.3	PE
CD45	BD Biosciences	30-F11	APC
SCA-1	BD Biosciences	D7	V500

Table S1.
List of used primary or secondary antibodies, with supplier and dosage
List of forward and reverse primers
List of used antibodies for FACS analysis
